# Research on the Multi-Degree-of-Freedom Programmable Lighting Method

**DOI:** 10.3390/s25175525

**Published:** 2025-09-05

**Authors:** Dianwu Ren, Jian Zhang, Zeng Peng, Haodong Shi, Dongpeng Yang, Songzhou Yang, Jingrui Sun, Yu Zhang, Bin Zhao, Taiyang Ren, Lu Wang, Yangyang Zou, Ke Zhang, Jiabo Lv

**Affiliations:** 1 School of Opto-Electronic Engineering, Changchun University of Science and Technology, Changchun 130022, China; 2 Opto-Electronic Measurement and Control Instrumentation, Jilin Province Engineering Research Center, Changchun 130022, China; 3 Key Laboratory of Opto-Electronic Measurement and Optical Information Transmission Technology, Ministry of Education, Changchun 130022, China; 4 Jilin Provincial Key Laboratory of Space Opto-Electronic Technology, Changchun University of Science and Technology, Changchun 130022, China; 5 School of Artificial Intelligence, Changchun University of Science and Technology, Changchun 130022, China; 6 State Key Laboratory of High Power Semiconductor Laser of Changchun University of Science and Technology, Changchun 130022, China; 7 Jilin Province Key Laboratory of Measuring Instrument and Technology, Jilin Institute of Metrology, Changchun 130022, China

**Keywords:** programmable lighting, spectral aberration compensation, wavelength scanning, intensity encoding, spectral modulation

## Abstract

To address the limitations of unsound physical models and the lack of compensation mechanisms in existing multi-degree-of-freedom programmable lighting methods, we proposed a novel multi-degree-of-freedom programmable lighting approach. The maximum deviation of single-wavelength spectral distribution curves before and after compensation was reduced by 2 times, and energy distribution uniformity was improved by 19.42 times. The wavelength scanning, intensity encoding, and broadband target spectral modulation performances were verified. The spectral modulation errors for CIE standard illuminants A and $D_{65}$ were −1.78% and −0.86%, respectively. This research lays the foundation for high-precision optical detection and analysis, enabling applications in biomedical and material fields.

## 1. Introduction

Programmable lighting technology surpasses the performance limitations of traditional optical system resolution [1] by enabling the synergistic modulation of multiple physical parameters. This capability supports the development of a new paradigm for optical systems [2]. At its core, programmable lighting technology enables the realization of high-precision dynamic coupling of wavelength [3], intensity [4], and time dimensions [5,6]. Functioning as the “intelligent optical engine” of modern optical imaging systems, this technology holds transformative potential in the field of biomedical dynamic imaging [7,8,9]; however, its ultimate performance is limited by its capacity to compensate for environmental perturbations and the depth of theoretical modeling of multi-physical field coupling mechanisms.

Programmable lighting technology primarily evolved from the development of static illumination modulation technology. In 2005, MacKinnon et al. introduced a spectrally programmable light source based on a digital micromirror device (DMD) [10,11,12] to achieve broad-spectrum target spectral simulation; however, its spectral resolution was limited to 10 nm. In 2013, Love et al. developed a full-frame programmable filter [13] capable of modulating more than 100 independent spectral channels. Despite its capability, implementation of the wavelength scanning mode remains challenging owing to the non-parallel alignment of DMD arrays with spectral channels. In 2016, Luo D et al. enhanced the architecture by designing a programmable light source that combines DMD and dispersive prisms [14], thereby achieving wide-band target spectral modulation across a wavelength range of 480–700 nm. With the development of static lighting modulation technology, recent research has been increasingly focused on algorithms. In 2023, Liu [15] reduced the spectral modulation error to below ±4.996% for color temperatures ranging from 3000 K to 11,000 K using a proportional–integral-derivative (PID) control algorithm. Building upon this, Yun [16] further reduced the spectral modulation error to ±3.5% through a backpropagation PID (BP-PID) control algorithm. These studies focus on improving the spectral modulation accuracy of static wide-band targets, but fail to systematically establish a spectral modulation model, thereby breaking through the physical limitations of dynamic modulation. With the development of programmable lighting technology, dynamic characteristic modulation has emerged as a focal point of research, accompanied by increasingly complex engineering challenges. In 2010, Clarke et al. [17] proposed the Fourier-domain programmable optical processor, capable of pulsed spectral modulation at multiple wavelengths; however, the system reliability was compromised due to its reliance on high-precision optical components. In 2016, Hirai [18] developed a multicolor projection system that can theoretically achieve a spectral resolution of 5 nm and simulate arbitrary spectral curves under time series. However, it was limited to the modulations of 4 to 6 basic colors. In 2019, Gu et al. [19] employed a DMD to shape femtosecond pulses, achieving rectangular, sawtooth, triangular, and other spectral profiles. In the same year, Yan et al. developed an all-digital optical frequency comb [20], which enabled the spectral modulation of an optical frequency comb with programmable comb spacing. However, the design was limited by the DMD pixel-level diffraction effects, which led to nonuniform comb intensity distribution, with the standard deviation exceeding 25%. In 2021, Mühleis et al. [21] designed a programmable spectral shaping system based on grating light valve technology, spanning 355–1200 nm for simulating the AM1.5G solar spectrum; however, the spectral resolution ranges from 7–15 nm. In 2024, Schenkel et al. [22] introduced a metrological-grade spectral source based on an optical parametric oscillator, achieving ultra-high-resolution and precision spectral output in the range of 2.2–3.9 μm, albeit at the cost of greatly increased system complexity. These bottlenecks essentially stem from the fact that optical–machine–electrical dynamic perturbations have not been quantified and modeled, and there is a lack of multi-physical field coupling models. At the same time, the wavelength and energy distribution patterns on the DMD cannot be clearly understood, resulting in modulation deviation compensation remaining at the feedback level.

Driven by advancements in imaging technologies such as computational optics, the demand for programmable lighting techniques in the medical field [23,24,25] has become increasingly prominent, revealing the shortcomings of existing techniques. In 2023, Sun et al. developed an unsupervised adaptive-coded illumination Fourier planar microscope [26] based on a physical neural network. By exploiting the physically scalable characteristics of light-emitting diode arrays, the system enhances data acquisition and computational efficiency by extending the physical neural network. In 2024, Wu et al. [27] developed a lensless on-chip three-dimensional microscope based on wavelength-scanning Fourier plane diffraction tomography using a wavelength-scanning system with narrow-band spectroscopy; however, this approach is limited by the spectral spacing of 1–11 nm, which affects the continuity and completeness of the reconstruction results. In the same year, as an alternative to the conventional mechanical scanning method, Skarsoulis et al. [28] introduced a simplified fiber endoscopic stack imaging system using a single-mode fiber for wavelength scanning. Although this confirmed the advantages of multi-wavelength imaging, it was only able to reconstruct binary images at three wavelengths.

In summary, programmable lighting technology has achieved significant progress and across key performance indexes, including wavelength scanning, intensity encoding, and wideband target spectral modulation. However, current research remains primarily focused on system design based on ideal theory and experimental verification based on engineering experience, with little research conducted on the construction of physical models for programmable lighting technology. This study identifies that the limitations of current multi-degree-of-freedom programmable lighting systems are attributed to inadequate physical modeling and the lack of an effective compensation mechanism, resulting in poor image quality and accuracy. To address these issues, in this study, we proposed a comprehensive multi-degree-of-freedom programmable lighting system architecture, which is based on a lighting model that incorporates the multi-physical coupling of a light source, spectral modulation optical system, DMD, beam mixing system, and homogeneous projection system. The proposed approach includes a systematic analysis of the spatial–spectral characteristics of a tungsten halogen lamp and the dispersion energy distribution behavior of the DMD. A compensation mechanism was developed to correct spectral distortion and nonlinear energy distribution, incorporating an integrating sphere and double-Gaussian synergistic transmission model. The spectral modulation optics and homogeneous projection systems were designed to compensate for spectral distortion and nonlinear energy distributions and validate the performance of wavelength scanning, intensity encoding, and wideband target spectral modulation. Furthermore, the proposed architecture facilitated quantitative comparison with existing techniques. Overall, this study offers a physically comprehensive and engineering solution to facilitate the integration of spectral analysis, computational optics, and medical diagnostics.

## 2. Coupled Modeling of Multi-Physical Properties for Multi-Degree-of-Freedom Programmable Lighting

**Figure 1:**
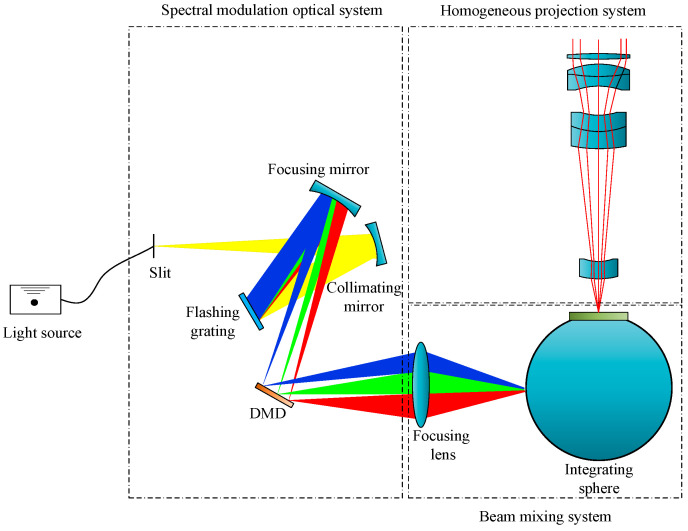
Overall structure and layout of the multi-degree-of-freedom programmable lighting system.

The topology of the multi-degree-of-freedom programmable lighting system comprises five principal components: a light source, a spectral modulation optical system, a DMD, a beam mixing system, and a homogeneous projection system. The light source employed in this study was a tungsten halogen lamp. The spectral modulation optical system features a crossed Czerny–Turner optical path. The beam mixing system integrates a focusing lens with an integrating sphere, whereas the homogeneous projection system utilizes a double-Gaussian projection lens. The overall structure and layout of the multi-degree-of-freedom programmable lighting system is shown in Figure 1.

The broad-spectrum beam emitted from the tungsten halogen lamp sequentially passes through the slit, collimating mirror, flashing grating, and focusing mirror within the spectral modulation optical system, and culminating in the formation of a dispersive spectral distribution in the DMD. Following compensation and modulation by the DMD, the light is directed through the focusing lens into the integrating sphere for spectral mixing and homogenization. The resulting output is then projected by the homogeneous projection system. Current research on multi-degree-of-freedom programmable lighting systems has been primarily concentrated on engineering aspects such as optical design [29,30], while lacking comprehensive models that capture the coupled multi-physical properties of the system. Notably, spectral distortion [31] and nonlinear energy distribution [32]—inherent to spectral modulation optics—remain insufficiently addressed. Therefore, based on multi-physical coupling, the present study proposed a multi-degree-of-freedom programmable lighting model that combines optical, mechanical, and electronic domains.

### 2.1. Modeling the Spatial–Spectral Distribution of the Light Source to the Slit

The radiation spectrum of a tungsten halogen lamp approximates that of blackbody radiation [33]. However, the spatial intensity of the light spot is a two-dimensional Gaussian distribution [34], and its spatial–spectral distribution characteristic function $S_{0}(\lambda ,\;x,\;y)$ is expressed as follows:

(1) $$S_{0}(\lambda ,\;x,\;y)=P_{0}(\lambda )\cdot e^{-\frac{x^{2}+y^{2}}{2\sigma_{s}^{2}}}$$

where $P_{0}(\lambda )$ is the spectral power distribution of the tungsten halogen lamp, λ is the wavelength of light, (*x*, *y*) are the spatial coordinates of light rays radiating the spectrum of the light source, $\sigma_{S}$ is the Gaussian radius of the light source, $P_{0}(\lambda )\propto \lambda^{-4}e^{-hc/(\lambda k_{B}T)}$ is the blackbody radiation approximation, h is Planck’s constant, c is the speed of light, $k_{B}$ is Boltzmann’s constant, and T is the blackbody temperature (the equivalent temperature of the tungsten halogen lamp). The slit opening function is a rectangular function [35], at which point the slit transmission function $T_{slit}(x,\;y)$ is expressed as

(2) $$T_{slit}(x,\;y)=rect\left(\frac{x}{w_{x}}\right)\cdot rect\left(\frac{y}{w_{y}}\right)$$

where $w_{x}$ and $w_{y}$ are the opening widths of the slit in the *x*- and *y*-directions (unit: mm). The light field distribution $E_{slit}(x,\;y,\;\lambda )$ after spatial filtering by the slit is calculated as follows:

(3) $$E_{slit}(x,\;y,\;\lambda )=S_{0}(\lambda ,\;x,\;y)\cdot T_{slit}(x,\;y)$$

where $S_{0}(\lambda ,\;x,\;y)$ is the spatial–spectral distribution of the light source, and $T_{slit}(x,\;y)$ is the transmission function of the slit.

### 2.2. Modeling of Spectral Dispersion and Energy Distribution from Slit to DMD Surface

The beam passing through the slit is irradiated onto the flashing grating. Considering the universality of the material, the grating equation λ(x) expanded to the third-order nonlinear term [36] can be expressed as follows:

(4) $$\lambda (x)=\lambda_{0}+k_{1}x+k_{3}x^{3}$$

where $k_{1}=\frac{d}{m_{g}R_{g}}\cos\;\beta_{0},\;k_{3}=-\frac{d}{6m_{g}{R_{g}}^{3}}\sin\;\beta_{0}\cdot (3{R_{g}}^{2}-f_{g}^{2})$, $\lambda_{0}$ is the center wavelength, $k_{1}$ is the linear dispersion coefficient, $k_{3}$ is the non-dispersive coefficient, d is the grating constant, $m_{g}$ is the grating diffraction level, $R_{g}$ is the grating radius of curvature, $\beta_{0}$ is the diffraction angle of the grating design, and $f_{g}$ is the grating focal length. Due to the orthogonality and completeness of Zernike polynomials, their intuitive correspondence to classical aberrations, and their natural adaptability to circular apertures, Zernike polynomials are used here to characterize the wavefront aberration W(x, y, λ) [37,38,39] as:

(5) $$W(x,\;y,\;\lambda )=\sum_{n_{t}=0}^{4}a_{n_{t}}(\lambda )Z_{n_{t}}\left(\frac{\sqrt{x^{2}+y^{2}}}{R_{sphere}}\right)$$

where $a_{n_{t}}(\lambda )$ is the Zernike polynomial coefficient, $Z_{n_{t}}$ is the Zernike polynomial, $R_{sphere}$ is the radius of curvature of the spherical mirror, and the coefficient $a_{n_{t}}=\frac{{R_{sphere}}^{n_{t}+1}}{4(n_{t}+1)}{\left(\frac{\lambda }{\lambda_{design}}\right)}^{n_{t}/2}$, where $\lambda_{design}$ is the design wavelength.

Considering the Fresnel integral of diffraction and aberration [40], at this point, the DMD surface light intensity distribution $I_{DMD}(x,\;y,\;\lambda )$ can be expressed as

(6) $$I_{DMD}(x,\;y,\;\lambda )={\left|F^{-1}\left[{\tilde{E}}_{slit}\cdot e^{i\frac{2\pi }{\lambda }W}\right]\right|}^{2}\cdot \eta_{g}(\lambda )$$

where $F^{-1}$ is the inverse Fourier transform operator, ${\tilde{E}}_{slit}$ is the complex amplitude distribution in the frequency domain after the incident light passes through the slit, $e^{i\frac{2\pi }{\lambda }W}$ is the phase factor introduced by the DMD micromirror modulation, and $\eta_{g}(\lambda )$ is the diffraction efficiency of the grating.

### 2.3. Modeling of Compensation Mechanisms for Spectral Distortion and Nonlinear Energy Distribution

The reflected field function $M_{pixel}(x,\;y,\;\lambda )$ of a single micromirror of the DMD can be expressed as follows:

(7) $$M_{pixel}(x,\;y,\;\lambda )=rect\left(\frac{x}{a}\right)\cdot e^{i\frac{4\pi }{\lambda }a\sin\theta }\cdot comb(x/a,y/a)$$

where a is the side length of a single micromirror, and θ is the tilt angle of the micromirror. At this point, the DMD modulation function is discretized M(x, y, λ, t) as

(8) $$M(x,\;y,\;\lambda ,\;t)=\sum_{m_{D},n_{D}}\Gamma_{m_{D}n_{D}}(t)\cdot M_{pixel}(x-m_{D}\Delta ,\;y-n_{D}\Delta ,\;\lambda )$$

where $\Gamma_{m_{D}n_{D}}(t)$ is the DMD modulation matrix indicating the state (0 or 1) of the $\left(m_{D},\;n_{D}\right)$-th micromirror at time t, and Δ is the spacing of micromirrors. At this time, the inverse mapping ${\hat{S}}_{in}(\lambda )$ of the spectral distortion is expressed as

(9) $${\hat{S}}_{in}(\lambda )=F^{-1}\left(I_{DMD}(x,\;y,\;\lambda ),\;\Gamma_{m_{D}n_{D}}(t)\right)$$

Let G(x, y, λ) be the flat-field energy distribution on the surface of the DMD and $h_{system}(x,\;y,\;\lambda )$ be the point spread function of the system comprising optical aberration, micromirror diffraction effect. The light intensity distribution on the surface of the DMD $I_{DMD}(x,\;y,\;\lambda )$ is expressed as follows:

(10) $$I_{DMD}(x,\;y,\;\lambda )=G(x,\;y,\;\lambda )⊗h_{system}(x,\;y,\;\lambda )$$

The energy gain attenuation coefficient is defined as $\alpha (x,\;y,\;\lambda )\in \left[0,1\right]$. By adjusting $\Gamma_{m_{D}n_{D}}$ and using an iterative method to minimize the objective function so that the actual light intensity distribution approximates the flat field energy distribution [41], the expression is:

(11) $$\Gamma_{m_{D}n_{D}}^{\left(k+1\right)}=\Gamma_{m_{D}n_{D}}^{k}+\mu \cdot \sum_{\lambda }\alpha (x_{m_{D}},\;y_{n_{D}},\;\lambda )\cdot \left[G(x_{m_{D}},\;y_{n_{D}},\;\lambda )-I(x_{m_{D}},y_{n_{D}},\lambda )\right]\cdot \frac{\partial I}{\partial \Gamma_{m_{D}n_{D}}}$$

where μ is the learning rate, and $\frac{\partial I}{\partial \Gamma_{m_{D}n_{D}}}$ can be obtained by calibration. At this point, the spectral distortion and nonlinear energy distribution compensation are incorporated into the following constrained optimization problem:

(12) $$\left\{\begin{array}{ll} \min_{M_{m_{D}n_{D}},\alpha } & {\left\|{\hat{S}}_{in}(\lambda )-S_{in}(\lambda )\right\|}^{2}+\beta {\left\|\alpha \cdot G-I\right\|}^{2}\\ s.t. & \Gamma_{m_{D}n_{D}}\in \{0,1\},\;\alpha \in [0,1] \end{array}\right.$$

where $S_{in}(\lambda )$ is the incident spectral distribution, and β is a weighting factor that balances spectral fidelity with energy uniformity.

### 2.4. Integrating Sphere-Homogeneous Projection Co-Transport Modeling

By considering that the integrating sphere–mixing light must go through many reflections, at this time, the radiative transfer equation $S_{out}(\lambda )$ of the integrating sphere is expressed as

(13) $$S_{out}(\lambda )=\frac{S{\left(\lambda \right)}_{port}}{1-\rho (\lambda )(1-f_{port})}$$

where $S{\left(\lambda \right)}_{port}$ is the spectral distribution in the integrating sphere, $f_{port}$ is the percentage of the integrating sphere port area, $f_{port}=A_{port}/A_{total}$, $A_{port}$ is the area of the integrating sphere port, $A_{total}$ is the total area of the inner wall of the integrating sphere, and ρ(λ) is the reflectivity of the inner wall of the integrating sphere.

Let the number of lenses of the double-Gaussian projection mirror set be $n_{p}$ and the focal length of each lens be $f_{i}$, then the double-Gaussian projection mirror set transfer function $H_{total}(k_{x},\;k_{y})$ is determined as follows:

(14) $$H_{total}(k_{x},\;k_{y})=\prod_{i=1}^{n_{p}}H_{i}(k_{x},\;k_{y})$$

where $H_{i}(k_{x},\;k_{y})$ is the transfer function of a single lens, $H_{i}(k_{x},\;k_{y})=\exp\left(-j\pi \lambda f_{i}(k_{x}^{2}+k_{y}^{2})\right)$, and $k_{x}$ and $k_{y}$ are the spatial frequency components. At this time, the light intensity distribution $I_{proj}(x,\;y)$ of the projection plane is obtained from the light intensity of the object plane $I_{obj}(x,\;y)$ that is modulated by the optical system, which can be expressed as follows:

(15) $$I_{proj}(x,\;y)=F^{-1}\left[F\{I_{obj}(x,\;y)\}\cdot H_{total}(k_{x},\;k_{y})\right]$$

## 3. Optical System Design and System Compensation

Tungsten halogen lamps [42] and DMDs [43] are well-established commercial products. The primary function of focusing lenses and integrating spheres is to mix and equalize light [44]. In this study, we chose the TP-WD-100W model tungsten halogen lamp, with a wavelength range of 300–2500 nm, and the DLP6500 model DMD, with a resolution of 1920 × 1080 and a pixel size of 7.56 μm. The focusing lens had an aperture of 26 mm, whereas the integrating sphere had a diameter of 24 mm and an opening ratio of 2%. Accordingly, this section focuses on the design of the spectral modulation optical system and the homogeneous projection system.

### 3.1. Design of the Spectral Modulation Optical System

**Figure 2:**
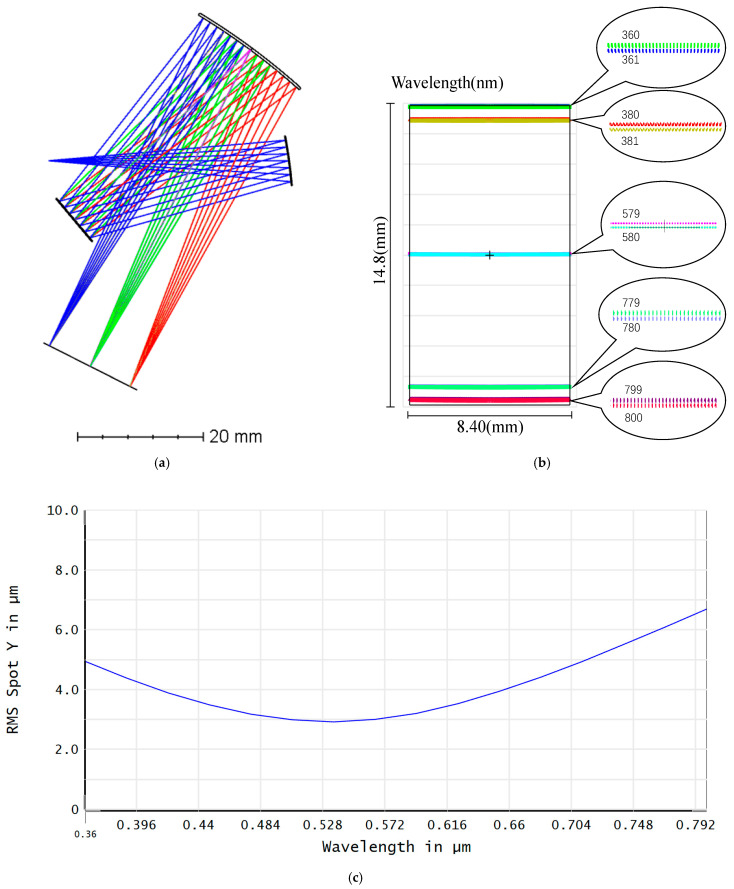
Optimization results of the spectral modulation optical system. (**a**) System diagram, (**b**) footprint diagram, and (**c**) full band *y*-axis Root Mean Square (RMS) radius.

The full visible light band ranging from 380–780 nm was chosen as the working band of the spectral modulation optical system to avoid the influence of edge wavelength on system performance. Therefore, the design band was extended from 360–800 nm. According to the grating diffraction equation

(16) $$d\left(\sin\;i-\sin\;\theta \right)=m\lambda$$

where the grating constant *d* = 1/*n*, *n* is the number of selected grating line pairs, and *m* is the diffraction level. The spectral resolution Δλ is characterized as

(17) $$\Delta \lambda =\frac{\omega \cos\;i}{\mu mn\bar{SM_{1}}}$$

where w denotes the slit width, $\bar{SM_{1}}$ is the distance between the slit and the collimating mirror, and μ is the correction factor for resolution. To achieve the separation of two distinct wavelengths, the diffraction level center must exceed the half-width diffraction angle of the grating. In this configuration, *m* is set to 1, *n* is set to 600 lines/mm. The incident and diffraction angles of the central wavelength light are denoted as ϕ. According to the grating equation, i is $25.38^{\circ }$, θ is $4.62^{\circ }$. According to ZEMAX (2024R1) the optimization results of the spectral modulation optical system are shown in Figure 2.

Figure 2 shows that the spectral resolution of the optimized spectral modulation optical system can be higher than 1 nm in the design band, and the Y-direction RMS radius of the spectral modulation optical system is less than 7 μm in the entire spectral range, which is smaller than the size of one microimage element of the DMD.

### 3.2. Design of the Homogeneous Projection System

**Figure 3:**
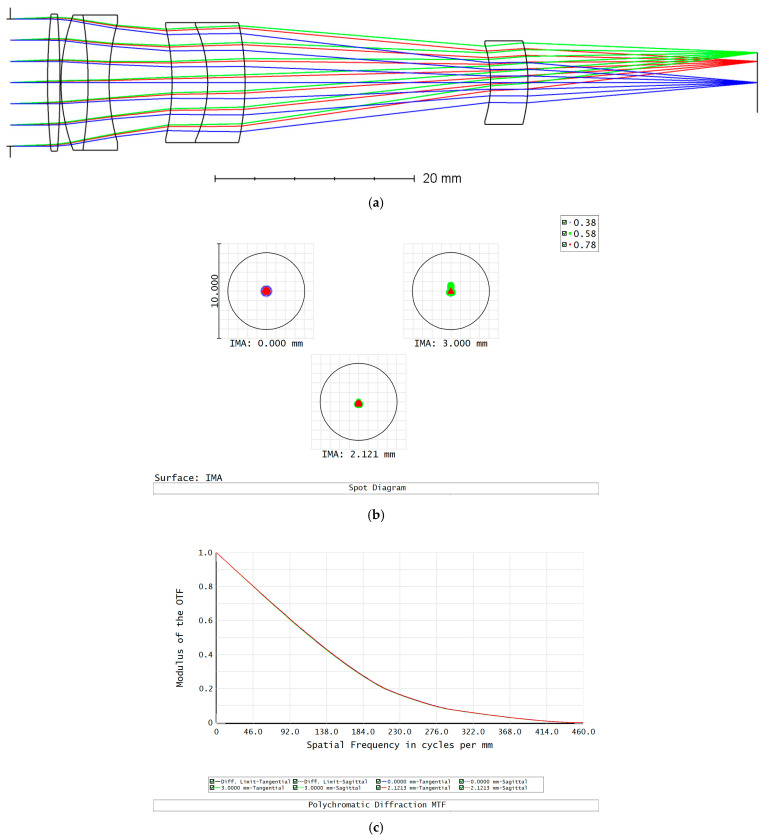
Results of the optimized homogeneous projection system design. (**a**) System diagram. (**b**) Spot diagram. (**c**) Modulation transfer function (MTF).

As the integrating sphere light source emits light uniformly [45], we employed a homogeneous projection system with a double Gaussian structure [46]. According to ZEMAX (2024R1), the results of the optimized homogeneous projection system design are shown in Figure 3.

As shown in Figure 3, the spot diagram of the optical system demonstrates good symmetry on the image plane and a controlled degree of dispersion. The RMS radius of the spot diagram of each field of view in the homogeneous projection system is smaller than the Airy radius, and the MTF reaches the diffraction limit, indicating high imaging quality.

### 3.3. Compensation for Spectral Distortion and Nonlinear Energy Distribution

**Figure 4:**
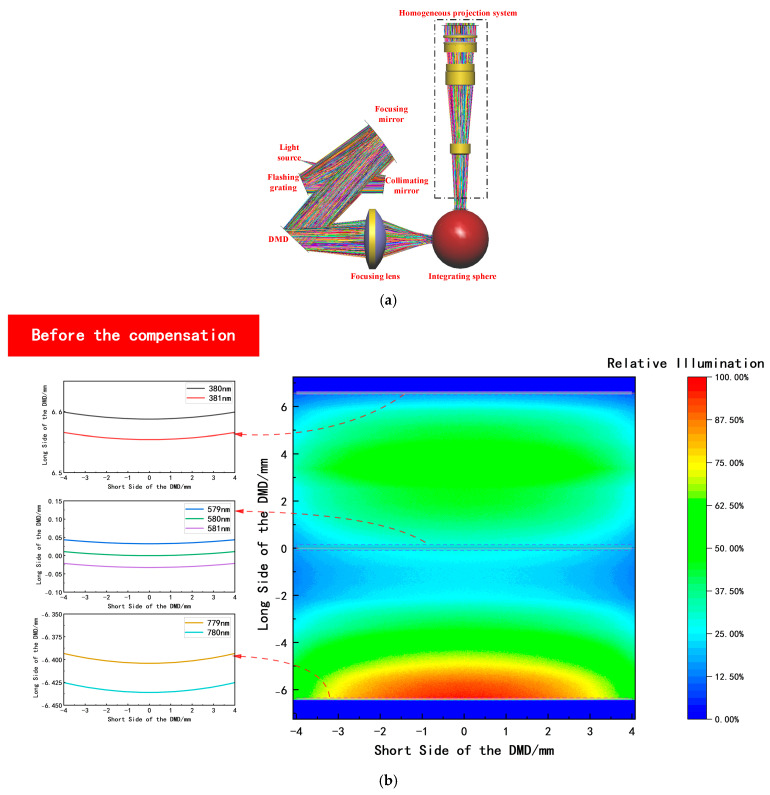

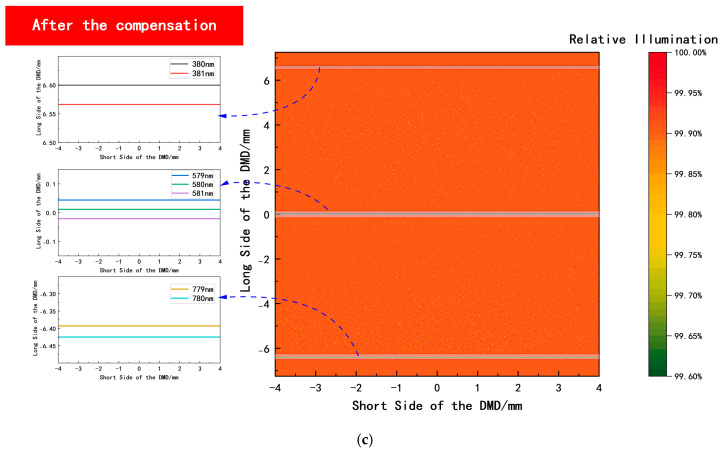
Effect of the compensation of spectral distortion and nonlinear energy distribution. (**a**) Simulation model of the multi-degree-of-freedom programmable lighting system. (**b**) Single-wavelength spectral distribution curves and energy distribution clouds before compensation. (**c**) Single-wavelength spectral distribution curves and energy distribution clouds after compensation.

According to LightTools (2023.03), a simulation model of the multi-degree-of-freedom programmable lighting system was established, as shown in Figure 4a. The resulting single-wavelength spectral distribution curves—corresponding to edge and center wavelengths of 380, 381, 579, 580, 581, 779, and 780 nm—along with associated the energy distribution clouds are shown in Figure 4b,c. These results depict the spectral and energy distributions on the surface of DMD arrays both before and after compensation. In practical applications, specific compensation results must be obtained based on the actual specific simulation parameters and statistical robustness during the dynamic modulation process of DMD.

With the aid of the simulation model of the multi-degree-of-freedom programmable lighting system, the single-wavelength spectral distribution curves before compensation can be obtained. Ideally, the single-wavelength spectral distribution curve after compensation should be a straight line parallel to the short side of the DMD [47], and the energy distribution cloud map should exhibit an approximately flat-topped energy distribution [48]. Therefore, to quantitatively evaluate the effectiveness of compensation, two metrics were employed: the maximum pixel deviation $E_{linearity}$ of the single-wavelength spectral distribution curve and the uniformity of energy distribution $E_{uniformity}$ [49]. The evaluation results before and after compensation are presented in Table 1. The formula of $E_{uniformity}$ is expressed as follows:

(18) $$E_{uniformity}=1-\left(\frac{E_{\max}-E_{\min}}{E_{\max}+E_{\min}}\right)\times 100\%$$

where $E_{\max}$ is the maximum irradiance of the spot, and $E_{\min}$ is the minimum irradiance of the spot.

## 4. Performance Verification and Comparison

### 4.1. Performance Multi-Degree-of-Freedom Programmable Lighting System

Performance verification is conducted from three perspectives: wavelength scanning, intensity encoding, and wide-band target spectral modulation.

#### 4.1.1. Wavelength Scanning

**Figure 5:**
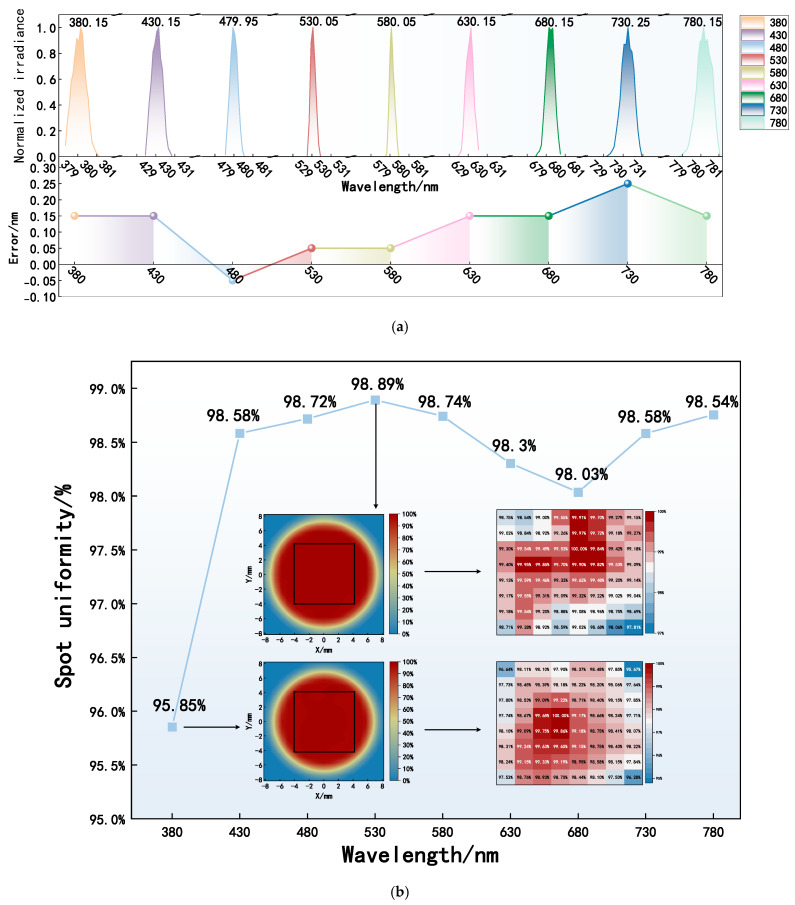
Wavelength scanning performance results. (**a**) Output peak wavelength spectral curves and peak wavelength error distributions. (**b**) Spot uniformity at different peak wavelengths.

The controlled, multi-degree-of-freedom programmable lighting system performed wavelength scanning at the 380–780-nm range with a peak interval of 50 nm. The output peak wavelength spectral curves and peak wavelength error distributions are shown in Figure 5a, and spot uniformity at different peak wavelengths is illustrated in Figure 5b.

As shown in Figure 5, the spectral curves of the peak wavelengths exhibit an overall distribution that is nearly symmetrical. However, in the vicinity of the peak wavelengths, different distribution patterns are observed. Specifically, the peak wavelength error at 480 nm exhibits a negative deviation, while the peak wavelength errors at other wavelengths exhibit positive deviations. The overall peak wavelength errors range between −0.05 nm and 0.25 nm. Based on the spot size, a 8 mm × 8 mm rectangular area was selected for local analysis of spot uniformity. Spot uniformity across different peak wavelengths shows a trend of initial growth followed by a gradual leveling off from 380 nm to 780 nm. Spot uniformity across different peak wavelengths ranged from 95.85–98.89%, with the lowest uniformity observed at 380 nm and the highest at 530 nm. In addition, except for the peak wavelength of 380 nm, the uniformity of all other wavelengths exceeds 98%. This is because 380 nm is at the edge of the system’s wavelength range, and the optical performance of the optical material decreases [50] at around 380 nm, ultimately affecting the energy distribution of the spot.

#### 4.1.2. Intensity Encoding

**Figure 6:**
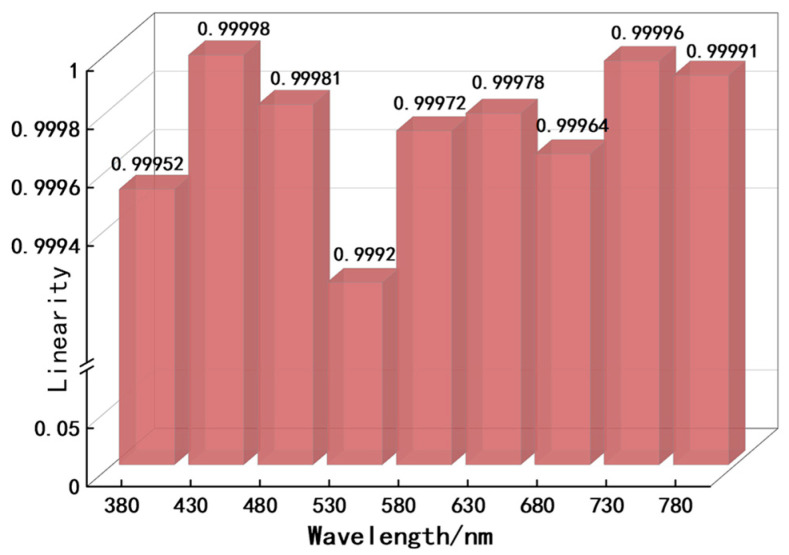
Linearity for different peak wavelengths.

Based on the wavelength scanning mode, intensity encoding was performed at wavelengths ranging from 380–780 nm with a peak interval of 50 nm. The linearity of the intensity encoding was measured using the R-square value [51] for different peak wavelengths, and the results are shown in Figure 6.

As shown in Figure 6, the peak wavelengths with the maximum and minimum linearities of intensity were 430 nm and 530 nm, respectively, and the linearity intervals ranged between 0.9992 and 0.99998, indicating good linearity.

#### 4.1.3. Wide-Band Target Spectral Modulation

**Figure 7:**
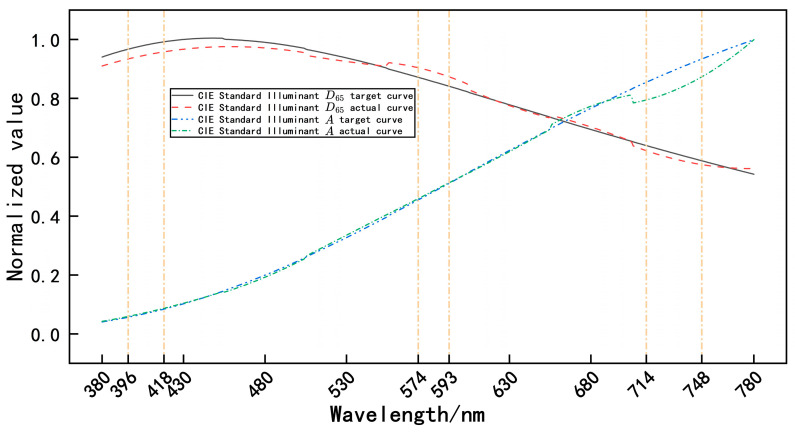
Results of specific target spectral profile modulation.

The common Commission Internationale de l’Eclairage (CIE) standard illuminant A [52], with a color temperature of 2856 K, and the CIE standard illuminant $D_{65}$ [53], with a color temperature of 6504 K, were selected for wide-band target spectral modulation. The spectral modulation accuracy $E_{total}$ [54] in the selected band was used to evaluate spectral modulation performance, and the formula is expressed as follows:

(19) $$E_{total}=\frac{\int_{380}^{780}S_{modulation}(\lambda )d\lambda -\int_{380}^{780}S_{objective}(\lambda )d\lambda }{\int_{380}^{780}S_{objective}(\lambda )d\lambda }$$

where $S_{modulation}$ is the spectral distribution after modulation, and $S_{objective}$ is the broadband target spectral distribution. Spectral modulation was performed using the fuzzy PID-based control algorithm [55], and the results of specific target spectral profile modulation are shown in Figure 7.

In Figure 7, the horizontal axis represents wavelength (nm), and the vertical axis represents normalized energy values. Figure 7 shows both the target curve and actual curve for the CIE standard illuminant A, as well as the target curve and actual curve for the CIE standard illuminant D_65_. The deviation between the curves represents the simulation accuracy. As shown in Figure 7, the $E_{total}$ of the CIE standard illuminant A was −1.78% in the 714–748-nm range. This deviation occurred because the normalized value of the simulated curve in this range approached its maximum and fell near the edge of the designed modulation bandwidth. Consequently, the simulated curve of the CIE standard illuminant A displayed a noticeable discrepancy from the standard curve, although it remained relatively close to the target curve. Similarly, the $E_{total}$ of the CIE standard illuminant $D_{65}$ was −0.86% in the range of 396–418 nm and 574–593 nm. As with Illuminant A, the normalized value of the simulation curve was close to the maximum value and fell near the edge of the designed modulation band. Furthermore, the simulation curve of CIE standard illuminant $D_{65}$ demonstrated noticeable discrepancy from the standard curve, although it remained relatively close to the target curve.

### 4.2. Comparison of Domestic and International Studies

This study demonstrates superiority in performance indexes compared with related studies. Specifically, the peak wavelength error in the wavelength scanning mode was improved by a factor of 35 to 75 compared with the grating light valve system developed by Mühleis [21] et al., and by a factor of 10 to 22 compared with the wavelength scanning system developed by Wu [27] et al. In the intensity encoding mode, the linearity of the output light intensity outperformed that of the optical frequency comb system developed by Yan [20] et al. In the spectral modulation mode, the wide-band target spectral modulation capability was comparable to that achieved by Yun [16] et al. using a fuzzy BP-PID, whereas the spectral shaping performance of the proposed system surpassed that of the pulse shaping system proposed by Gu [19] et al. Furthermore, this study achieves the synergistic control of wavelength scanning, intensity encoding, and wide-band target spectral modulation through a single system architecture, integrating three spectral modulation dimensions that could previously only be controlled independently. This simplifies the system structure and breaks through the technical limitations of similar studies that only support a single function. A comparison of tasks and indexes between the present and previous studies is presented in Table 2.

## 5. Conclusions and Outlook

In this study, we proposed a multi-degree-of-freedom programmable lighting system, along with establishing a comprehensive system architecture composed of spectral modulation optical and homogeneous projection systems. By establishing a multi-degree-of-freedom programmable lighting model with multi-physical coupling and systematically analyzing the spatial–spectral characteristics of the tungsten halogen lamp and the DMD dispersion energy distribution law, a compensation mechanism was formulated to address spectral distortion and nonlinear energy distribution, supported by the construction of an integrating sphere–double Gaussian synergetic transmission model. By considering the crossed Czerny–Turner optical path and the double-Gaussian lens structures as initial configurations, the spectral modulation optical and homogeneous projection systems were optimized. The resulting spectral modulations system achieved a spectral resolution of higher than 1 nm across the 360–800-nm range, with an RMS radius of less than 7 μm in the Y-direction—which is smaller than the size of the micro-mirror element of a single DMD. For the projection system, the RMS spot radius in each field of view of the homogeneous projection system was below the Airy radius, with the modulation transfer function closely approaching the diffraction limit. A simulation model of the multi-degree-of-freedom programmable lighting system was established, enabling the compensation of both spectral distortion and nonlinear energy distribution of the multi-degree-of-freedom programmable lighting system. The results showed that compensation reduced the maximum deviation in single-wavelength spectral distribution curves by a factor of 2 while improving energy distribution uniformity by a factor of 19.42. The performance of multi-degree-of-freedom functionality was validated across three aspects: wavelength scanning, intensity encoding, and wide-band target spectral modulation. Within the 380–780-nm range, the maximum peak wavelength error was 0.25 nm, spot uniformity exceeded 95.85%, intensity encoding linearity was greater than 0.9992, and the system supports the accurate spectral modulation of target-specific spectra. The spectral modulation errors for the CIE standard illuminant A and the CIE standard illuminant $D_{65}$ were −1.78% and −0.86%, respectively.

In addition, this study overcomes the inherent functional limitations of conventional single-purpose spectral modulation systems by integrating all three core functions—wavelength scanning, intensity encoding, and wide-band target spectral modulation—within a unified architecture. Furthermore, the performance of wavelength scanning and intensity encoding of the proposed system was significantly better than that of existing single-function architectures, whereas the accuracy of wide-band target spectral modulation was comparable to relevant international standards. This technology provides critical technical support for precision spectral analysis, dynamic optical detection, and adaptive imaging. Particularly in the biomedical field, this study provides a solid theoretical foundation and technological platform for innovative applications: leveraging its high-precision, high-speed spectral modulation capabilities, it is anticipated to drive the development of cutting-edge technologies such as non-invasive blood glucose monitoring, early cancer biomarker screening, and real-time intraoperative tissue identification. It may also offer unprecedented dynamic spectral insights for brain function imaging and drug metabolism studies, ultimately delivering breakthrough tools for precision medicine and personalized health management. In the future, the system architecture can be further optimized by adopting closed-loop or open-loop and closed-loop hybrid composite control strategies, and introducing distributed control and modular design to improve the scalability and stability of the system. Moreover, the system can be integrated into confocal microscopes and other advanced optical platforms. This enables the construction of new-generation tools for super-resolution imaging and dynamic spectral modulation, which is achieved through advanced neural network algorithms. These innovations would support cutting-edge research in areas such as in vivo cell observation and high-resolution material micro-region analysis.

## Figures and Tables

**Table 1:** Comparison of the Effects Before and After Compensation.

	$E_{linearity}$/pixel	$E_{uniformity}$/%
Before Compensation	4	5.14
After Compensation	2	99.80
Enhancement Factor	2	19.42

**Table 2:** Comparison of Tasks and Indexes Between the Present and Previous Studies.

Literature		Implementation Functions	Main Technical Indicators
Grating light valve spectral shaping system (Mühleis et al., 2021) [21]		AM1.5G solar spectrum simulation	Spectral resolution: 7 to 15 nm
Wavelength scanning system (Wu et al., 2024) [27]		Single-wavelength spectral scanning	Spectral resolution: 1 to 11 nm
All-digital optical frequency comb (Yan et al., 2019) [20]		Optical frequency comb modulation with programmable comb spacing	Adjustable comb spacing, and strength nonuniformity standard deviation of >25%
DMD femtosecond pulse shaping (Gu et al., 2019) [19]		Pulse shape spectral shaping	Rectangle/jagged/triangle spectrum generation
BP-PID control of spectral simulation (Yun, 2024) [16]		Wide-band target spectral simulation	Spectral modulation error of less than ±3.5%
This study	Wavelength scanning mode	Wavelength continuous scan output	Wavelength error of <0.2 nm and uniformity of >95.85
	Intensity encoding mode	Intensity linear modulation	Linearity of >0.9992
	Wide-band target spectral modulation mode	Arbitrary target spectral shaping	Spectral modulation error of less than ±1.78%

## Data Availability

The raw data supporting the conclusions of this article will be made available by the authors upon request.
